# Milk exosomes: beyond dietary microRNAs

**DOI:** 10.1186/s12263-017-0562-6

**Published:** 2017-06-22

**Authors:** Janos Zempleni

**Affiliations:** Department of Nutrition and Health Sciences, University of Nebraska-Lincoln, Lincoln, NE USA

**Keywords:** Exosomes, Extracellular vesicles, Milk

## Abstract

Extracellular vesicles deliver a variety of cargos to recipient cells, including the delivery of cargos in dietary vesicles from bovine milk to non-bovine species. The rate of discovery in this important line of research is slowed by a controversy whether the delivery and bioactivity of a single class of vesicle cargos, microRNAs, are real or not. This opinion paper argues that the evidence in support of the bioavailability of microRNAs encapsulated in dietary exosomes outweighs the evidence produced by scholars doubting that phenomenon is real. Importantly, this paper posits that the time is ripe to look beyond microRNA cargos and pursue innovative pathways through which dietary exosomes alter metabolism. Here, we highlight potentially fruitful lines of exploration.

## Advancing the field of milk vesicles

All multicellular and unicellular organisms communicate with their environment through extracellular vesicles (EVs) [1–4]. Healthy cells produce and secrete two major classes of EVs: exosomes are about 100 nm in size and are derived from endosomes in the multivesicular body; microvesicles are between 200 and 1000 nm in size and are formed by blebbing of the plasma membrane [4].

This paper focuses on exosomes due to their essential roles in cell-to-cell communication through shuttling a variety of cargos among tissues [1, 5–9]. Exosomes and their cargos have been implicated in virtually all physiological and pathological conditions [10–18]. Cargos include various species of coding and non-coding RNAs, proteins, and lipids [8, 19–22]. The loading of exosomes with cargos is not a random process but involves sorting mechanisms that favor some cargos over others [21, 23, 24]. Exosomes may deliver their cargos over short distances to receptor cells adjacent to the exosome-secreting donor cell, or cargos may be delivered to receptor cells in distant tissues [1, 8, 9, 11, 25].

Evidence suggests that exosomes and their cargos are not only derived from endogenous synthesis but can also be obtained from dietary sources, particularly bovine and human milk. Milk exosomes are of particular interest because they constitute a scalable source of exosomes for drug loading and delivery (bovine), the essential role of (human) milk in infant nutrition, and the large volume of (bovine) milk and other dairy consumed by Americans [26, 27]. This paper has a focus on the biological activity of exosomes and their cargos from bovine milk in non-bovine species but also discusses other dietary sources of exosomes where appropriate.

Bovine milk exosomes enter human and rat intestinal cells and human endothelial cells by endocytosis [28, 29], enter circulating immune cells [20], and accumulate in peripheral tissues [26, 30]. A report that bovine milk exosomes can be detected in virtually all peripheral tissues was based on studies that lacked vehicle controls [26]; the widespread distribution of bovine milk exosomes among tissues in non-bovine species awaits confirmation. Prime candidates are tissues rich in resident immune cells such as liver, spleen and lung, and the site of absorption in the small intestine [30–33]. Encapsulation of labile cargos in exosomes confers a mechanism of protection against harsh conditions in the intestinal tract such as low pH in the stomach and against exposure to enzymes such as RNases and proteases [34, 35]. Collectively, it is possible that milk exosomes deliver bioactive cargos to hosts following oral administration.

Unfortunately, the rate of discovery in the field of dietary exosomes and their cargos has been slowed down by a continuing controversy whether a particular class of cargos, microRNAs, is delivered across species boundaries and elicit biological effects, or dietary microRNAs in body fluids are too low to elicit effects or might be assay artifacts (reviewed in [36]). These discussions are ongoing and, in the author’s opinion, have not lead to a satisfactory resolution of the dispute whether dietary microRNAs have biological activity. The author proposes that momentum is building in support of the theory that dietary microRNAs are bioavailable and alter gene expression across species boundaries, based on reports from a large number of independent laboratories including ours [37–50]. Strong arguments in favor of the bioavailability of dietary RNAs include the following. (1) Exogenous microRNAs were detected by RNA sequencing (RNA-seq) in human plasma and breast milk [38–40]. (2) There is consensus that bovine milk exosomes are bioavailable [26, 28–30, 33]. (3) Feeding a diet depleted of bovine milk exosomes and microRNAs caused a more than 60% decrease in plasma microRNAs compared with controls [39]. (4) Some genetically modified organisms utilize synthetic microRNA analogs, small interfering RNAs (siRNAs), to achieve gene knockdown in pests [51] (e.g., DvSnf7 siRNA in Monsanto’s Smart Stax Pro corn; [52]. The siRNAs in these organisms are biologically active (i.e., kill pests upon absorption). (5) RNAs encapsulated in bovine milk exosomes survive harsh conditions such as low pH [34] and digestion under simulated gastrointestinal tract conditions [35].

Some critical voices remain. Concerns were raised regarding “ineffective microRNA delivery of oral microRNAs” and the possibility of sample contamination [53–55]. One of these reports was based on the analysis of samples in which the dry ice was sublimated during shipping to the investigators’ laboratory and therefore should be discounted [56]. Title et al. employed an interesting cross-fostering strategy and detected only trace amounts of miR-375 in the plasma of miR-375 knockout pups fostered to wild-type dams [55]. The authors disregarded the possibility that, upon intestinal absorption, miR-375 binds to transcript targets in the intestinal mucosa and liver, followed by rapid degradation (the classical “first passage elimination” effect), which is consistent with miRNA “sponge” use in microRNA research [57, 58], and our observation that the majority of milk exosomes accumulate in the intestinal mucosa and liver [30, 33]. Title et al. could not have been aware of a later report that the sequence motif, (A/U)(C_2-4_)(A/U), is essential for miRNA packaging into exosomes [59]; the motif is missing in miR-375 although other motifs might exist.

We propose that while these somewhat myopic deliberations continue, the field of dietary exosomes needs to explore new pathways by which dietary exosomes may elicit phenotypes. Here, we highlight a few examples. First, a substantial portion of exosomes in bovine milk escapes absorption and enters the large intestine [30]. When considering that microorganisms communicate with their environment through EVs [3], it would be worthwhile to study the effects of dietary EV intake on the gut microbiome. Our preliminary data suggest that feeding a diet deplete of vesicles from bovine milk causes changes in the gut microbiome in mice [60]. Second, exosomes contain a variety of bioactive lipids, proteins, and non-coding RNAs other than microRNAs [8, 19–22]. Evidence is emerging that exosomes may deliver some of these compounds to target tissues [61]. Third, RNAs including microRNAs may bind to Toll-like receptors to regulate immune responses [62]. It may be worthwhile to explore whether RNAs in milk exosomes also bind to Toll-like receptors. Fourth, there is the possibility that the mere interactions of exosome with the cell surface may alter metabolism, as proposed by Askenase and co-workers [9]. Finally, we caution against making the assumption that milk exosomes and their cargos will necessarily travel to the same destination. Our ongoing studies suggest that bovine milk exosomes and their RNA cargos travel to distinct tissues [33]. Analytical preparations of exosomes and microvesicles may be contaminated with other classes of EVs or contain sub-populations from the same class of EVs with distinct biological functions [63]. Investigators need to examine protocols used for vesicle preparations to assess the identity of vesicles in a given study and the levels of rigors applied by them [6].

Studies of milk vesicles and their roles in human metabolism are an exciting line of research. We hope that the exploration of new pathways will rapidly advance this line of research in the future. This will not only be important for human nutrition but also for the delivery of drugs by bovine milk exosomes [26]. It will be important not to lose sight of the tremendous potential of dietary exosomes in the light of the sweltering controversy surrounding their microRNA cargos.

## Abbreviations

EVs: Extracellular vesicles
